# 5-hydroxytryptamine has an endothelium-derived hyperpolarizing factor-like effect on coronary flow in isolated rat hearts

**DOI:** 10.1186/s12929-015-0149-8

**Published:** 2015-06-16

**Authors:** Ching-Chia Chang Chien, Ming-Jai Su

**Affiliations:** Institute of Pharmacology, College of Medicine, National Taiwan University, 11F No.1 Sec.1, Ren-ai Rd, Taipei, 10051 Taiwan

**Keywords:** Serotonin, 5-HT, Nitric oxide, NO, Endothelium-derived hyperpolarization factor, EDHF, Coronary flow, Rat, NO-independent

## Abstract

**Background:**

5-hydroxytryptamine (5-HT)-induced coronary artery responses have both vasoconstriction and vasorelaxation components. The vasoconstrictive effects of 5-HT have been well studied while the mechanism(s) of how 5-HT causes relaxation of coronary arteries has been less investigated. In isolated rat hearts, 5-HT-induced coronary flow increases are partially resistant to the nitric oxide synthase inhibitor Nω-Nitro-L-arginine methyl ester (L-NAME) and are blocked by 5-HT_7_ receptor antagonists. In the present study, we investigated the role of 5-HT_7_ receptor in 5-HT-induced coronary flow increases in isolated rat hearts in the absence of L-NAME, and we also evaluated the involvement of endothelium-derived hyperpolarizing factor (EDHF) in 5-HT-induced coronary flow increases in L-NAME-treated hearts with the inhibitors of arachidonic acid metabolism and the blockers of Ca^2+^-activated K^+^ channels.

**Results:**

In isolated rat hearts, 5-HT and the 5-HT_7_ receptor agonist 5-carboxamidotryptamine induced coronary flow increases, and both of these effects were blocked by the selective 5-HT_7_ receptor antagonist SB269970; in SB269970-treated hearts, 5-HT induced coronary flow decreases, which effect was blocked by the 5-HT_2A_ receptor blocker R96544. In L-NAME-treated hearts, 5-HT-induced coronary flow increases were blocked by the phospholipase A_2_ inhibitor quinacrine and the cytochrome P450 inhibitor SKF525A, but were not inhibited by the cyclooxygenase inhibitor indomethacin. As to the effects of the Ca^2+^-activated K^+^ channel blockers, 5-HT-induced coronary flow increases in L-NAME-treated hearts were inhibited by TRAM-34 (intermediate-conductance Ca^2+^-activated K^+^ channel blocker) and UCL1684 (small-conductance Ca^2+^-activated K^+^ channel blocker), but effects of the large-conductance Ca^2+^-activated K^+^ channel blockers on 5-HT-induced coronary flow increases were various: penitrem A and paxilline did not significantly affect 5-HT-induced coronary flow responses while tetraethylammonium suppressed the coronary flow increases elicited by 5-HT.

**Conclusion:**

In the present study, we found that 5-HT-induced coronary flow increases are mediated by the activation of 5-HT_7_ receptor in rat hearts in the absence of L-NAME. Metabolites of cytochrome P450s, small-conductance Ca^2+^-activated K^+^ channel, and intermediate-conductance Ca^2+^-activated K^+^ channel are involved in 5-HT-induced coronary flow increases in L-NAME-treated hearts, which resemble the mechanisms of EDHF-induced vasorelaxation. The role of large-conductance Ca^2+^-activated K^+^ channel in 5-HT-induced coronary flow increases in L-NAME-treated hearts needs further investigation.

## Background

5-hydroxytryptamine (5-HT) has both vasoconstrictive and vasodilating effects on coronary arteries [1, 2]; injecting low doses of 5-HT into normal human coronary arteries induces vascular dilations, but at high doses 5-HT injections cause vasoconstrictions [1]. The vasoconstrictive effect of 5-HT on coronary arteries is mostly mediated by 5-HT_2A_ receptor, and to a lesser extent by the activation of 5-HT_1B_ receptor [3, 4]. The knowledge about the vasoconstrictive effects of 5-HT on coronary arteries has been applied to develop treatments of coronary artery diseases [3].

5-HT_7_ receptor is the latest identified subtype of 5-HT receptors [5]. It couples to Gs protein and induces cAMP accumulation when activated [6], and in rat glomerulosa cells the activation of 5-HT_7_ receptor increases calcium influx via T-type Ca^2+^ channels by raising adenylyl cyclase activity [7]. Activation of 5-HT_7_ receptor has been reported mediating 5-HT-induced relaxation in isolated dog coronary arteries [8] and 5-HT-induced coronary flow increases in Nω-Nitro-L-arginine methyl ester hydrochloride (L-NAME)-treated rat hearts [9]. The functional role of 5-HT_7_ receptor in human coronary arteries has not been reported, but the mRNA expression of 5-HT_7_ receptor in human coronary vasculature has been identified [4].

The mechanism(s) of how 5-HT mediates coronary artery relaxation remains controversial. In dog coronary arteries, both endothelium-dependent [10] and endothelium-independent [8] vasodilating effects of 5-HT have been reported. In rat, coronary flow increases/coronary artery relaxation effect of 5-HT has been proposed to be endothelium-dependent [11, 12] and nitric oxide (NO)-dependent [13]; however, prostacyclin (PGI_2_)-dependent [14] and nitric oxide synthase (NOS) inhibitor-resistant [9] components of this effect have also been reported.

Endothelium-derived hyperpolarizing factor (EDHF) is a putative factor that mediates endothelium-dependent vasorelaxation [15, 16]. It induces vasodilation by hyperpolarizing the membrane potential of smooth muscle cells, which effect consequently prevents the activation of Ca^2+^ channels and reduces Ca^2+^ influx [17]. Although the mechanism(s) and the end effector(s) of EDHF-induced vasorelaxation remain controversial [18], activations of Ca^2+^-activated K^+^ channels by arachidonic acid metabolites synthesized by endothelial lipoxygenases (LOXs) and cytochrome P450s are known to be involved in EDHF-mediated vascular responses [18, 19].

In our previous study, we found that 5-HT-induced coronary flow increases in isolated rat hearts have a L-NAME-resistant component [9], and the 5-HT-induced coronary flow increases in L-NAME-treated hearts are blocked by 5-HT_7_ receptor antagonists [9]; however, the role of 5-HT_7_ receptor activation in the NO-dependent component of 5-HT-induced coronary flow increases was left unevaluated, and the mechanism(s) of the L-NAME-resistant component of the coronary flow-increasing effect was not studied. In the present study, we investigated the role of 5-HT_7_ receptor in 5-HT-induced coronary flow increases in the absence of L-NAME in isolated rat hearts by using selective 5-HT receptor agonist and antagonist, and we also evaluated the role of EDHF in 5-HT-induced coronary flow increases by testing effects of blockers of arachidonic acid metabolism [18, 20] and Ca^2+^-activated K^+^ channels [16] on 5-HT-induced coronary flow increases in L-NAME-treated hearts.

## Methods

### Animals

Adult male Sprague Dawley rats aged 2–3 months (weighting 250–350 g) were purchased from BioLasco Co. (Yilan, Taiwan). Animals were kept in the Laboratory Animal Center of National Taiwan University (Taipei, Taiwan) until the day of experiment. Rats were given *ad libitum* access to water and food. All animal procedures were performed according to the *Guide for the Care and Use of Laboratory Animals* of the National Institutes of Health, and followed the guidelines of the Animal Welfare Act. The animal studies were approved with a certificate number 20110073 by the Institutional Animal Care and Use Committee of the College of Medicine, National Taiwan University (Taipei, Taiwan).

### Chemicals and solution

5-HT, adenosine, histamine, indomethacin, tetraethylammonium chloride (TEA), L-NAME, and dimethyl sulfoxide (DMSO) were purchased form Sigma-Aldrich (St. Louis, Missouri, USA). SB269970, R96544, 5-carboxamindotryptamine (5-CT), TRAM-34, and UCL1684 were purchased from Tocris Bioscience (Bristol, United Kingdom). Quinacrine, penitrem A, paxilline, and SKF525A were purchased from Cayman Chemical Co. (Ann Arbor, Michigan, USA).

The perfusion solution used in the present study was modified Tyrode’s solution (in mM): 119.7 NaCl, 23.8 NaHCO_3_, 5.6 Glucose, 1.2 CaCl_2_, 1.1 MgCl_2_, 0.3 NaH_2_PO_4,_ and 5.0 KCl. In the experiments testing the effects of the inhibitors of arachidonic acid metabolism and the blockers of Ca^2+^-activated K^+^ channels on 5-HT-induced coronary flow increases, L-NAME 10 μM was added to the perfusion solution and existed throughout the experiments.

### Preparation of isolated perfused rat hearts

Rats were intraperitoneally injected with pentobarbital 50 mg/kg and heparin 800 IU/kg. 15 min later, rats were killed by cervical dislocation and hearts were quickly removed and mounted on the Langendorff apparatus (ADInstruments, Castle Hill, Australia).

Hearts were perfused with modified Tyrode’s solution at constant pressure (70 mmHg). The perfusion solution was kept at 37 °C and well gassed with carbogen (O_2_ 95 % and CO_2_ 5 % mixture).

Tissue around the sinoatrial node was trimmed to slow down the spontaneous heart rate. Stimulating electrodes were placed on the left atrium, and recording electrodes were attached to the apex to record electric cardiogram (EKG). Hearts were constantly paced at 250 b.p.m throughout experiment with stimulations generated by a stimulator with stimulation length of 2 ms and an interval of 240 ms. Hearts were then left to stabilize for 30 min before subjected to experiment. The perfusion pressure, coronary flow, and EKG were demonstrated and recorded continuously on computer using PowerLab program (ADInstruments, Castle Hill, Australia).

### Effects of the 5-HT receptor antagonists on 5-HT-induced coronary flow responses in isolated perfused hearts

Hearts were prepared as described above. After 30 min stabilization, hearts were treated with vehicle, SB269970 0.3 μM (a selective 5-HT_7_ receptor antagonist [21]), or SB269970 0.3 μM + R96544 0.3 μM (a selective 5-HT_2A_ receptor antagonist [22]) for 4 min; 5-HT 0.3 and 1 μM were then added into perfusion solution cumulatively to test effects of these antagonist(s) on 5-HT-induced coronary responses. These concentrations of 5-HT were chosen on the basis of our preliminary observations, in which 5-HT-induced coronary flow responses are not stable at concentrations higher than 1 μM (data not shown).

**Fig. 1:**
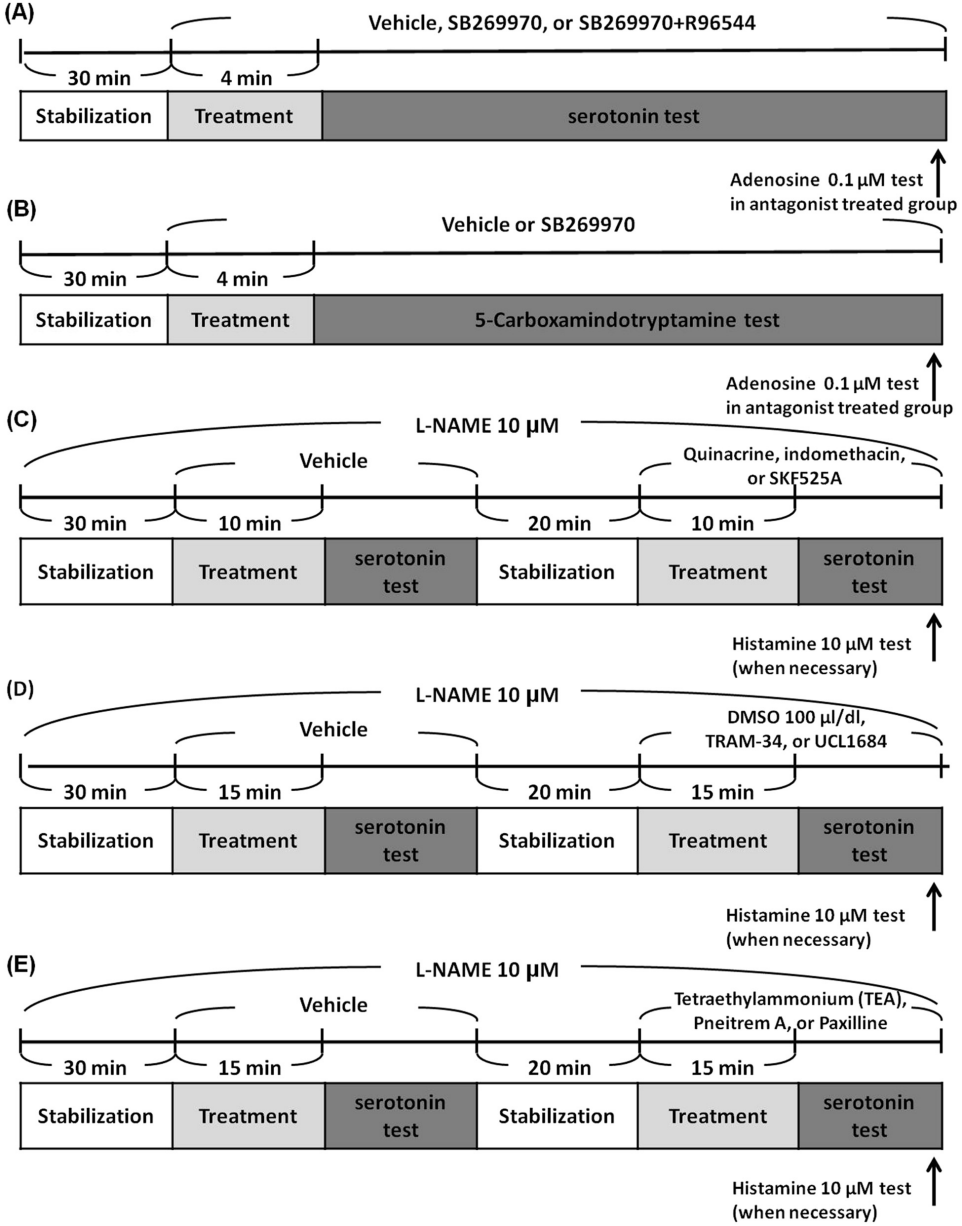
Schemes of experiment protocols

5-HT-induced coronary flow increases have been reported as NO-dependent [12], so in the antagonist(s)-treated groups we added adenosine 0.1 μM, which is also an NO/endothelium-dependent [23] vasodilator of rat coronary arteries, to test the viability of endothelium and the activity of NOS at the end of experiments. The protocol is summarized in Fig. 1a.

### Effect of SB269970 on 5-CT-induced coronary flow responses in isolated perfused hearts

Hearts were prepared as described above. After stabilization, hearts were treated with vehicle (DMSO 1 μl/dl) or SB269970 0.3 μM for 4 min, and then 10 nM and 30 nM of 5-CT, a selective and potent 5-HT_1B/7_ receptor agonist [24], were added into the perfusion solution in a dose-accumulative manner in the presence of vehicle or SB269970 to test the effect of the 5-HT_7_ receptor antagonist on 5-CT-induced coronary flow increases. Adenosine 0.1 μM was added into the perfusion solution at the end of experiment to test the viability of endothelium and the activity of NOS if 5-CT failed to elicit coronary flow increase in the presence of SB269970. The protocol is summarized in Fig. 1b.

### Effects of the inhibitors of arachidonic acid metabolism on 5-HT-induced coronary flow increases in L-NAME-treated rat hearts

Isolated perfused hearts were prepared and the experiments were performed with L-NAME-containing perfusion solution as mentioned above.

After 30 min stabilization, hearts were treated with vehicle (DMSO 10 μl/dl) for 10 min, and coronary flow responses to 5-HT 0.3 and 1 μM were tested in the presence of vehicle. After the tests of coronary flow responses to 5-HT were established, each heart was perfused with fresh L-NAME-containing solution for 20 min to remove the effect of 5-HT and re-stabilize. Hearts were treated with 1 μM quinacrine (phospholipase A_2_ (PLA_2_) inhibitor [15]), 3 μM SKF525A (cytochrome P450s inhibitor [25]), or 10 μM indomethacin (cyclooxygenase (COX) inhibitor [26]) for 10 min, respectively, and then coronary flow responses to 5-HT were tested again in the presence of these treatments. Histamine is an endothelium-dependent vasodilator of rat coronary arteries [27, 28], which relaxes smooth muscle in a NO-independent manner [29], so we added histamine 10 μM to test the viability of the coronary arteries at the end of experiments if 5-HT failed to elicit coronary flow responses. The protocol is summarized in Fig. 1c.

### Effects of the Ca^2+^-activated K^+^ channel blockers on 5-HT-induced coronary flow responses in L-NAME-treated hearts

Hearts were prepared and perfused with L-NAME 10 μM as described above. After 30 min equilibration, each heart was perfused with vehicle for 15 min, and then coronary flow responses to 5-HT, at concentrations of 0.3 and 1 μM, were tested in the presence of vehicle. In the TRAM-34- and UCL1684-treated groups, DMSO 100 μl/dl was used as vehicle control in the control tests; in the penitrem A- and paxilline-treated groups, DMSO 10 μl/dl was used as vehicle control in the control tests. The vehicle of TEA was the perfusion solution, so we just perfused the hearts for 15 min as vehicle treatment. After the first serial of 5-HT tests, hearts were then perfused with fresh L-NAME-containing perfusion solution for 20 min to re-equilibrate. After re-equilibration, hearts were perfused with TRAM-34 10 μM (selective blocker of intermediate-conductance Ca^2+^-activated K^+^ channel [30]), UCL1684 3 μM (selective blocker of small-conductance Ca^2+^-activated K^+^ channel [31]), penitrem A 1 μM [32], paxilline 2 μM [33] (both selective blockers of large-conductance Ca^2+^-activated K^+^ channel), or TEA 300 μM for 15 min, and then coronary flow responses to 5-HT 0.3 and 1 μM were tested again in the presence of these treatments. At the end of each experiment, histamine 10 μM was added into the perfusion solution to test the viability of coronary vasculature if 5-HT failed to elicit coronary flow increases in the presence of the treatments.

To validate the design of the experiments, we tested the effect of DMSO 100 μl/dl, which was the vehicle we used in the UCL1684-treated and TRAM-34-treated groups and also the largest quantity of DMSO we used in this article, on 5-HT-elicited coronary flow responses by the same protocol used in testing the effects of Ca^2+^-activated K^+^ channel blockers. The protocols are summarized in Fig. 1d and e.

### Statistic analysis

Data were expressed as mean ± SD. Intra-group analyses were performed with one-way ANOVA followed with Bonferroni post-tests. Inter-groups differences were analyzed by 2-ways ANOVA followed by Bonferroni post-tests. Variables from the adenosine tests and the histamine tests were not used when performing ANOVA and Bonferroni post-tests. P < 0.05 was considered to be statistically significant.

## Results

### Effects of the 5-HT receptor antagonists on 5-HT receptor agonist-induced coronary flow responses

**Fig. 2:**
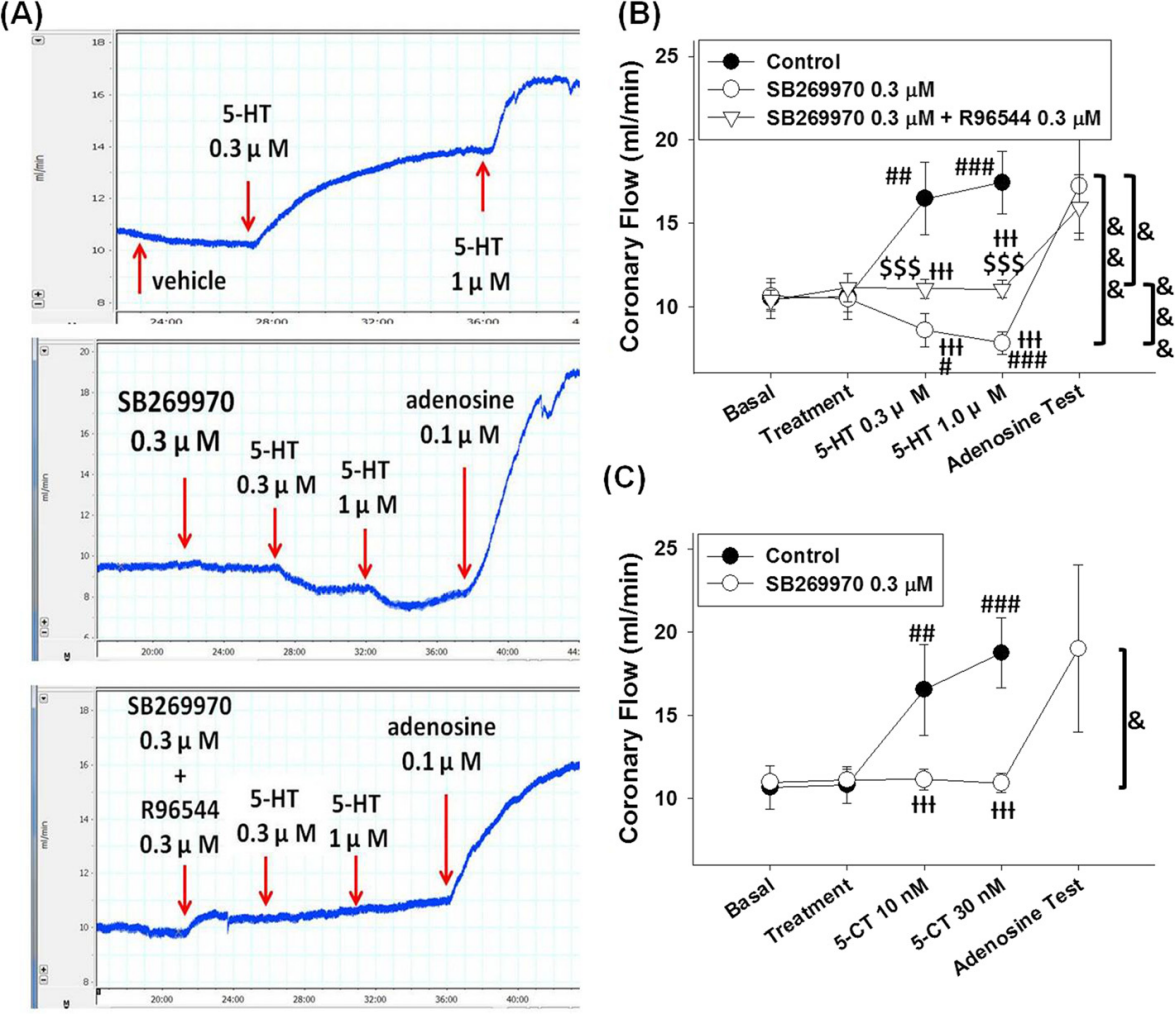
Effects of the 5-HT receptor antagonists on 5-HT-induced coronary responses in isolated rat hearts. **a** Typical traces of 5-HT-induced coronary flow responses in the presence of vehicle, SB269970, or the combination of SB269970 and R96544. **b** In isolated rat heart, 5-HT induced coronary flow increases, but in 5-HT_7_ receptor antagonist SB269970 0.3 μM-treated hearts, 5-HT caused decreases in coronary flow. In hearts treated with SB269970 0.3 μM and the 5-HT_2A_ antagonist R96544 0.3 μM, 5-HT did not elicit a coronary response. **c** The 5-HT_7_ receptor agonist 5-CT elicited coronary flow increases, and this effect was blocked by SB269970 0.3 μM. Data were expressed as the mean ± SD. #P < 0.05, ##P < 0.01, and ###P < 0.005 compared to the treatment; ƗƗƗP < 0.005 compared to the control group (one-way ANOVA followed by Bonferroni post-test); $$$P < 0.005 compared to the SB269970-treated group; &P < 0.05, &&&P < 0.005 between the compared groups (2-ways ANOVA followed by Bonferroni post-test)

Figure 2a shows the typical tracings of 5-HT-induced coronary flow responses in the presence of vehicle, SB269970, and combination of SB269970 and R96544.

As shown in Fig. 2b, 5-HT elicited coronary flow increases in the presence of vehicle in the control group (P < 0.0001 and F = 19.11, one-way ANOVA). Vehicle (DMSO 1 μl/dl) did not significantly alter coronary flow in the control group (basal value 10.52 ± 1.22 ml/min and after DMSO treatment 10.60 ± 1.37 ml/min); 5-HT significantly increased coronary flow to 16.48 ± 2.18 (P < 0.01 compared to treatment of vehicle) and 17.45 ± 1.87 ml/min (P < 0.005 compared to treatment of vehicle) at concentrations of 0.3 μM and 1 μM, respectively.

5-HT 0.3 and 1 μM induced coronary flow decreases in the presence of SB269970 0.3 μM (Fig. 2b) (n = 5; P < 0.0001 and F = 14.33, one-way ANOVA), which were significantly different from the responses in the control group (P = 0.0007 and F = 33.71, 2-ways ANOVA analyzed with the control group). Treatment with SB269970 0.3 μM did not significantly alter coronary flow (10.60 ± 1.37 ml/min compared to basal value 10.50 ± 1.22 ml/min); in the presence of SB269970 0.3 μM, coronary flow decreased to 8.59 ± 1.01 ml/min (P < 0.05 compared to treatment) and 7.82 ± 0.68 ml/min (P < 0.005 compared to treatment) after treatments of 5-HT 0.3 and 1 μM, respectively. At the end of experiment, addition of adenosine 0.1 μM caused an increase of coronary flow to 17.79 ± 2.74 ml/min in the presence of SB269970 and 5-HT.

As shown in Fig. 2a and b, 5-HT failed to elicit coronary flow response at doses of 0.3 and 1 μM in hearts treated with the combination of SB269970 0.3 μM and R96544 0.3 μM (n = 4; P = 0.345 and F = 1.22, one-way ANOVA). Treatment of SB269970 0.3 μM + R96544 0.3 μM slightly increased coronary flow to 11.15 ± 0.83 ml/min from the basal value of 10.38 ± 0.61 ml/min. In the presence of SB269970 and R96544, coronary flow was not significantly altered by 5-HT at concentrations of 0.3 and 1 μM (11.07 ± 0.58 ml/min and 11.07 ± 0.54 ml/min, respectively). At the end of experiment, addition of adenosine 0.1 μM increased coronary flow to 15.96 ± 1.94 ml/min. The 5-HT-induced coronary flow responses in SB269970 + R96544-treated group were significantly different from the SB269970-treated group (P = 0.0056 and F = 15.54, analyzed by 2-ways ANOVA with the SB269970 treated group) and the control group (P = 0.0138 and F = 11.82, 2-ways ANOVA analyzed with the control group).

5-CT is a potent 5-HT_1B/7_ receptor agonist with low affinity to the 5-HT_2A_ receptor (Ki value 633–2700 nM) [24]. As shown in Fig. 2c, 5-CT elicited coronary flow increases in the control group (n = 4; P < 0.0001 and F = 18, one-way ANOVA), and 5-CT-elicited coronary flow increases were blocked by SB269970 0.3 μM (P = 0.0169 and F = 12.4, 2-ways ANOVA). Treatment with vehicle did not elicit significant coronary flow response in the control group (10.83 ± 1.09 ml/min compared to basal value 10.67 ± 1.29 ml/min); in the presence of vehicle, 5-CT 10 nM induced a coronary flow increase to 16.54 ± 2.74 ml/min, and 5-CT 30 nM increased the coronary flow to 18.74 ± 2.11 ml/min (P < 0.01 and P < 0.005 compared to the vehicle treatment point, respectively). In the SB269970-treated group, 5-CT failed to elicit coronary flow response at 10 and 30 nM (n = 3; P = 0.9573 and F = 0.1008, one-way ANOVA). Treatment of SB269970 0.3 μM did not significantly alter coronary flow (11.13 ± 0.63 ml/min compared to the basal value 10.99 ± 0.37 ml/min); however, it blocked the coronary flow-increasing effect of 5-CT. In the presence of SB269970 0.3 μM, 5-CT at 10 nM or 30 nM did not elicited significant coronary flow alteration (11.15 ± 0.63 ml/min and 10.94 ± 0.59 ml/min, respectively). In the precence of SB269970 0.3 μM and 5-CT 30 nM, addition of adenosine 0.1 μM increased coronary flow to 19.01 ± 5.03 ml/min at the end of experiment.

### Effects of the inhibitors of arachidonic acid metabolism on 5-HT-induced coronary flow increases in L-NAME-treated hearts

**Fig. 3:**
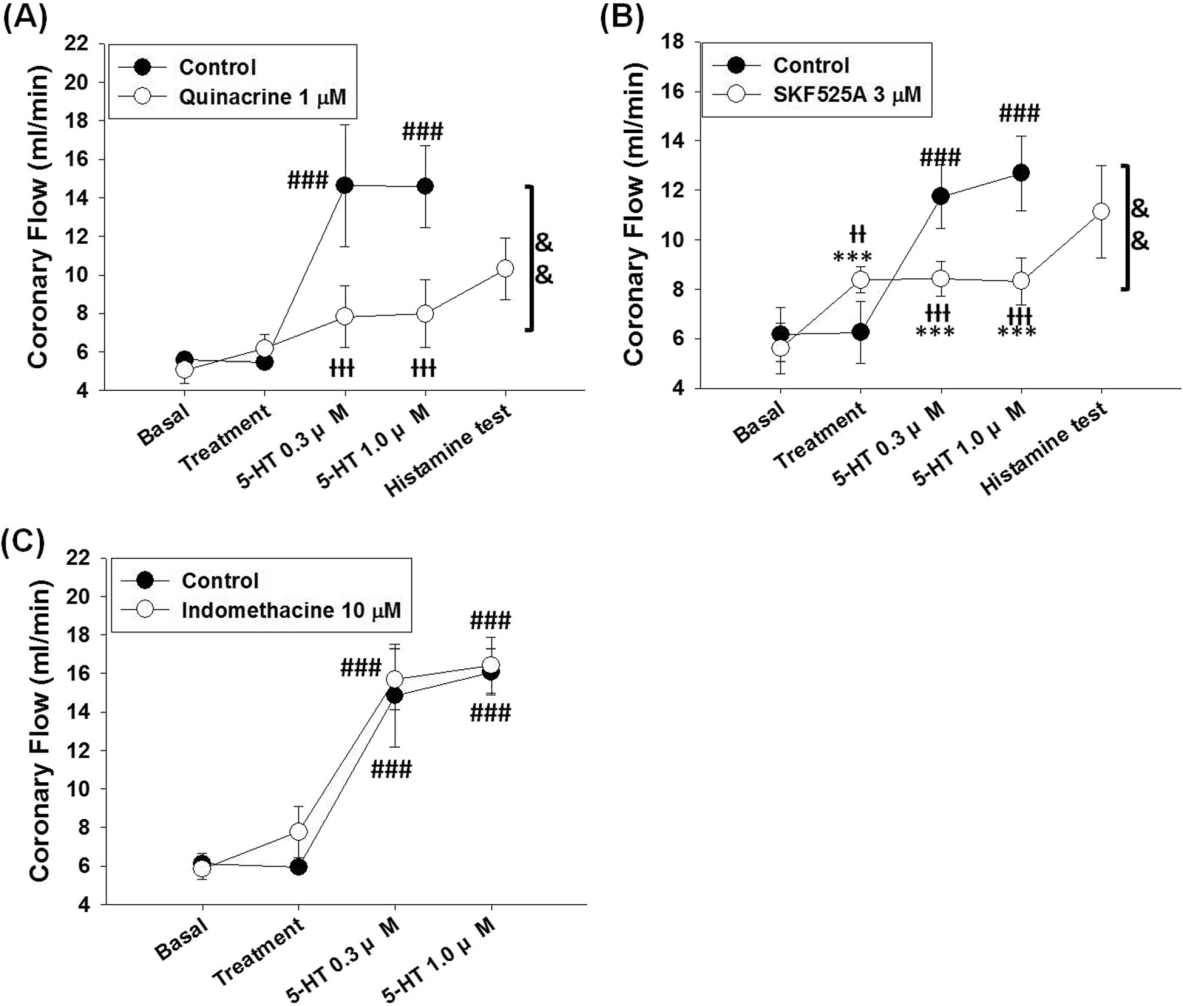
Effects of the inhibitors of arachidonic acid metabolism on 5-HT-induced coronary response in L-NAME-treated hearts. **a** Treatment with the PLA_2_ blocker quinacrine 1 μM slightly increased coronary flow, and after treatment of quinacrine, 5-HT-induced coronary flow increases were suppressed. **b** Treatment with the cytochrome P450 inhibitor SKF525A 3 μM significantly increased coronary flow, and in the presence of SKF525A, 5-HT-induced coronary flow increases were blocked. **c** Treatment of the COX inhibitor indomethacin 10 μM did not significantly influence 5-HT-induced coronary flow increase. Data were expressed as the mean ± SD. ***P < 0.005 compared to basal value; ###P < 0.005 compared to the treatment. ƗƗP < 0.01, ƗƗƗP < 0.005 compared to the control group (one-way ANOVA followed by Bonferroni post-test); &&P < 0.01 between the compared groups (2-ways ANOVA followed by Bonferroni post-test)

Quinacrine (Fig. 3a) significantly suppressed the 5-HT-induced coronary flow increases in L-NAME-treated rat hearts (n = 5; P = 0.0032 and F = 16.73, 2-ways ANOVA). In the control test, 5-HT significantly increased coronary flow (P < 0.0001 and F = 37.1, one-way ANOVA); the basal coronary flow value in the control test was 5.58 ± 0.41 ml/min after perfusion with L-NAME 10 μM for 30 min. After 10 min treatment with vehicle, coronary flow did not significantly change (5.45 ± 0.37 ml/min). Perfusion of 5-HT at concentrations of 0.3 and 1 μM significantly increased coronary flow to 14.63 ± 3.16 ml/min and 14.58 ± 2.13 ml/min (both P < 0.001 compared to the vehicle treatment), respectively. Treatment with quinacrine 1 μM slightly increased coronary flow to 6.16 ± 0.74 ml/min from 5.07 ± 0.68 ml/min; and after the treatment with quinacrine, 5-HT induced slight coronary flow increases at 0.3 μM (7.82 ± 1.62 ml/min) and 1 μM (7.98 ± 1.77 ml/min). The values of coronary flow at 5-HT 0.3 and 1 μM in the presence of quinacrine were significantly lower than those in the control test (both P < 0.001 compared to the corresponding doses in the control test). Histamine 10 μM increased the coronary flow to 10.31 ± 1.61 ml/min at the end of the experiment.

As shown in Fig. 3b, 5-HT-induced coronary flow increases were significantly suppressed in the presence of SKF525A 3 μM (n = 7; P = 0.008 and F = 9.738, 2-ways ANOVA). 5-HT induced significant coronary flow increases in the control test (P < 0.0001 and F = 50.89, one-way ANOVA); basal value of the control test was 6.16 ± 1.10 ml/min, and treatment with vehicle did not significantly alter coronary flow (6.25 ± 1.26 ml/min). 5-HT at concentrations 0.3 and 1 μM increased coronary flow to 11.74 ± 1.28 (P < 0.01) and 12.68 ± 1.50 (P < 0.005) ml/min, respectively. Coronary flow was significantly altered after treatment of SKF525A 3 μM and 5-HT (P < 0.0001 and F = 19.77, one-way ANOVA). SKF525A 3 μM increased coronary flow from the basal value of 5.60 ± 1.02 ml/min to 8.37 ± 0.54 ml/min (P < 0.005), which was proposed to be mediated by the release of PGI_2_ from endothelium [34]. In the presence of SKF525A, 5-HT did not significantly alter coronary flow at concentrations 0.3 and 1 μM (8.41 ± 0.70 and 8.33 ± 0.95 ml/min, respectively), and coronary flow after treatments of 5-HT 0.3 and 1 μM were significantly lower than the corresponding values in the control test (both P < 0.005). Histamine 10 μM increased coronary flow to 11.13 ± 1.85 ml/min in the histamine test at the end of experiment.

Treatment with indomethacin 10 μM (Fig. 3c) did not significantly affect 5-HT-induced coronary flow increases in L-NAME-treated hearts (n = 4; P = 0.3299 and F = 0.09741, 2-ways ANOVA). 5-HT elicited significant increases of coronary flow in the control test (n = 4; P < 0.0001 and F = 39.81, one-way ANOVA). Treatment with vehicle did not significantly increase the coronary flow (basal value 6.11 ± 0.57 ml/min and the value after vehicle treatment 5.94 ± 0.37 ml/min) in the control test. 5-HT increased coronary flow to 14.85 ± 2.67 and 16.08 ± 1.19 ml/min at concentrations 0.3 μM and 1 μM (both P < 0.001 compared to the vehicle treatment point), respectively. 5-HT elicited coronary flow increases in the presence of indomethacin 10 μM (P < 0.0001 and F = 64.01, one-way ANOVA). Treatment with indomethacin slightly increased coronary flow to 7.78 ± 1.32 min/ml from the basal value of 5.84 ± 0.52 ml/min. In the presence of indomethacin, 5-HT 0.3 μM and 1 μM increased the coronary flow to 15.65 ± 0.79 ml/min and 16.56 ± 0.73 ml/min (both P < 0.001 compared to corresponding points in the control test), respectively. Since the 5-HT-induced coronary flow responses were not significantly altered, we did not perform histamine test in this group at the end of the experiment.

### Effects of the Ca^2+^-activated K^+^ channel blockers on 5-HT-induced L-NAME-resistant coronary flow increases

**Fig. 4:**
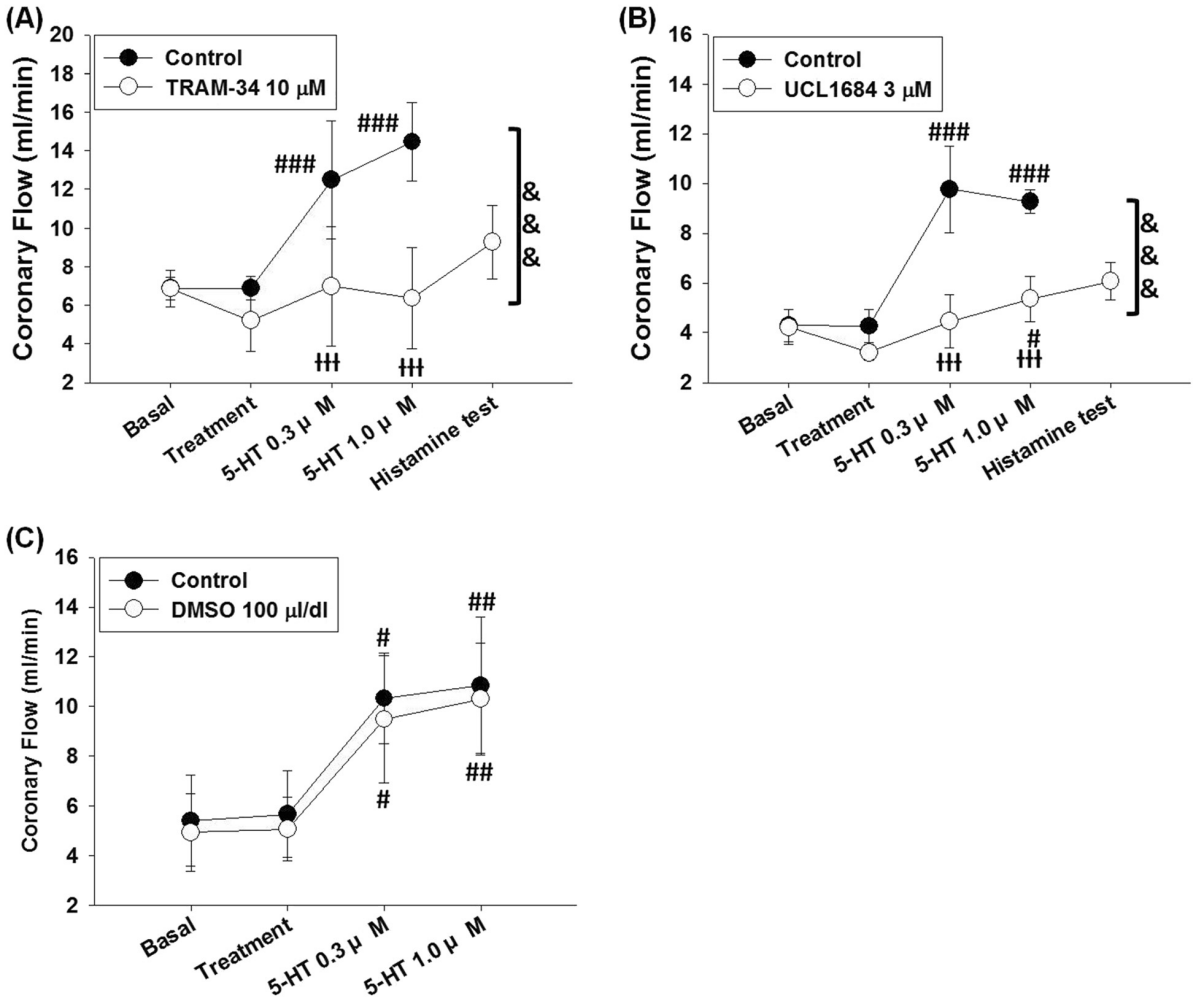
Effects of small- and intermediate-conductance Ca^2+^-activated K^+^ channel blockers on 5-HT-induced coronary response. In LNAME-treated hearts: **a**5-HT-induced coronary flow increases were significantly suppressed by TRAM-34, which is a selective blocker of intermediate-conductance Ca^2+^-activated K^+^ channel. **b** In hearts treated with the selective blocker of small-conductance Ca^2+^-activated K^+^ channel UCL1684 3 μM, 5-HT elicited slight coronary flow increase at 1 μM; but compared to the control test, 5-HT-induced coronary flow increases were significantly suppressed. **c** Coronary flow responses to 5-HT were not significantly altered after re-equilibration, prolonged perfusion with L-NAME, and repeated treatments of vehicle (DMSO 100 μl/dl). Data were expressed as the mean ± SD. #P < 0.05, ##P < 0.01, and ###P < 0.005 compared to the treatment (one-ways ANOVA followed by Bonferroni post-test). ƗƗƗP < 0.005 compared to the control group; &&&P < 0.001 between the compared groups (2-ways ANOVA followed by Bonferroni post-test)

As shown in Fig. 4a, treatment of TRAM-34 10 μM significantly suppressed 5-HT-induced coronary flow increases in L-NAME treated hearts (n = 5; P = 0.0047 and F = 15.03, 2-ways ANOVA). 5-HT elicited coronary flow increases in the control test (P < 0.0001 and F = 21.42, one-way ANOVA); treatment with vehicle (DMSO 100 μl/dl) did not significantly alter coronary flow (6.88 ± 0.61 ml/min compared to the basal value 6.84 ± 0.59 ml/min) in the control test, and 5-HT 0.3 and 1 μM increased coronary flow to 12.49 ± 3.04 ml/min (P < 0.001 compared to vehicle treatment) and 14.47 ± 2.05 ml/min (P < 0.001 compared to vehicle treatment), respectively. 5-HT failed to elicit coronary flow increases after treatment of TRAM-34 10 μM (P = 0.5857 and F = 0.6649, one-way ANOVA). Treatment of TRAM-34 10 μM slightly decreased coronary flow to 5.20 ± 1.59 ml/min from the basal value (6.86 ± 0.59 ml/min). In the presence of TRAM-34, 5-HT did not significantly alter coronary flow at 0.3 μM (6.99 ± 3.09 ml/min) or 1 μM (6.38 ± 2.63 ml/min). Coronary flow increased to 9.28 ± 1.90 ml/min in the histamine test at the end of experiment.

5-HT-induced coronary flow increases were significantly suppressed by UCL1684 3 μM (n = 4; P = 0.0036 and F = 21.33, 2-ways ANOVA) (Fig. 4b). In the control test, 5-HT induced significant coronary flow increases (P < 0.0001 and F = 35.55, one-way ANOVA). Treatment of vehicle (DMSO 30 μl/dl) did not cause significant alteration of coronary flow (5.66 ± 1.74 ml/min compared to the basal value 5.41 ± 1.82 ml/min). Coronary flow increased to 10.32 ± 1.82 ml/min and 10.84 ± 2.75 ml/min after perfusion with 5-HT at concentrations of 0.3 and 1 μM (both P < 0.001 compared to the vehicle treatment point), respectively. In the presence of UCL1684, 5-HT elicited slight increases of coronary flow (P = 0.0189 and F = 4.899, one-way ANOVA). Treatment of UCL1684 3 μM slightly reduced the coronary flow from the basal value of 4.28 ± 0.70 ml/min to 3.20 ± 0.32 ml/min. In the presence of UCL1684 treatment, 5-HT slightly increased coronary flow to 4.46 ± 1.08 ml/min at 0.3 μM, and 5-HT increased coronary flow to 5.37 ± 0.92 ml/min at 1 μM (P < 0.05 compared to the ULC1684 treatment point). The values of coronary flow at 5-HT 0.3 and 1 μM in the presence of UCL1684 are lower than the corresponding values in the control test (both P < 0.001). At the end of the experiment, addition of histamine 10 μM increased coronary flow to 6.07 ± 0.77 ml/min in the presence of UCL1684 and 5-HT.

As shown in Fig. 4c, repeated treatments of DMSO 100 μl/dl did not significantly alter 5-HT-induced coronary flow responses (n = 5; P = 0.6122 and F = 0.2782, 2-ways ANOVA). In the control test, 5-HT elicited coronary flow increases in the presence of DMSO 100 μl/dl (P = 0.0006 and F = 9.938, one-way ANOVA). The basal value of coronary flow was 5.41 ± 1.82 ml/min, and perfusion with DMSO 100 μl/dl for 15 min did not significantly alter coronary flow (5.66 ± 1.74 ml/min) in the control test. In the presence of DMSO 100 μl/dl, perfusion with 5-HT at concentrations of 0.3 and 1 μM increased coronary flow to 10.32 ± 1.82 ml/min (P < 0.05 compared to the vehicle treatment point) and 10.84 ± 2.75 ml/min (P < 0.01 compared to the vehicle treatment point), respectively. In the second test, 5-HT still elicited coronary flow increases (P = 0.0005 and F = 10.28, one-way ANOVA). Perfusion with DMSO 100 μl/dl, as in the control test, did not significantly alter coronary flow (5.06 ± 1.28 ml/min compared to basal value 4.93 ± 1.57 ml/min); 5-HT increased coronary flow to 9.48 ± 2.57 at 0.3 μM and to 10.30 ± 2.24 ml/min at 1 μM (P < 0.05 and P < 0.01 compared to DMSO 100 μl/dl treatment, respectively) in the presence of DMSO 100 μl/ml.

**Fig. 5:**
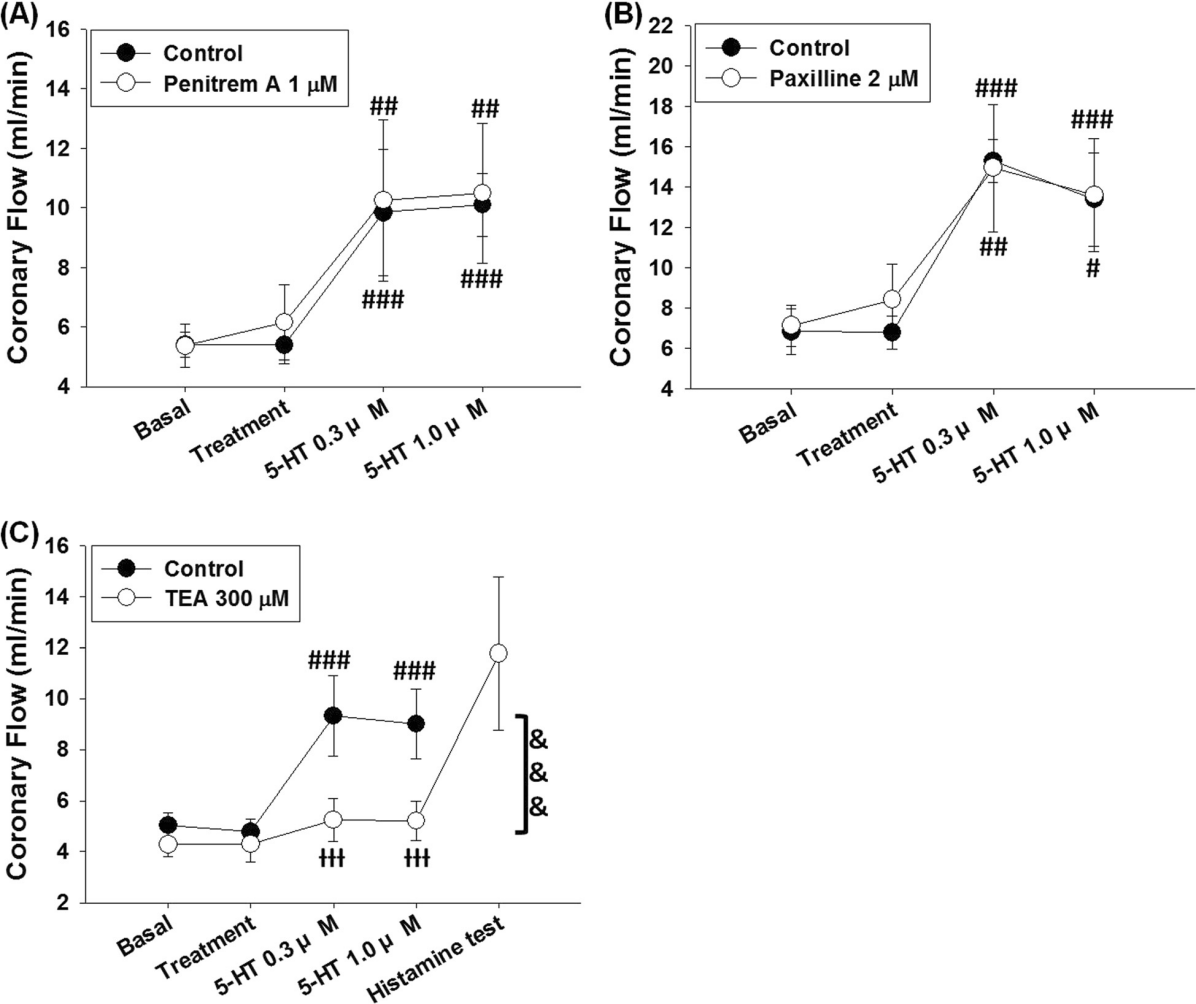
Effects of the large-conductance Ca^2+^-activated K^+^ channel blockers on 5-HT-induced coronary responses in L-NAME-treated hearts. **a** 5-HT-induced coronary flow increases were not significantly affected by penitrem A 1 μM. **b** Treatment of paxilline 2 μM did not significantly alter 5-HT-induced coronary flow increases. **c** 5-HT-induced coronary flow increases were significantly suppressed by treatment with TEA 300 μM. Data were expressed as the mean ± SD. #P < 0.05, ##P < 0.01, ###P < 0.005 compared to the treatment (one-ways ANOVA followed by Bonferroni post-test). ƗƗƗP < 0.005 compared to control group; &&&P < 0.005 between the compared groups (2-ways ANOVA followed by Bonferroni post-test)

Treatment of penitrem A 1 μM did not significantly altered 5-HT-induced coronary flow increases in L-NAME treated hearts (n = 7; P = 0.5661 and F = 0.3481, 2-ways ANOVA) (Fig. 5a). 5-HT elicited coronary flow increases in the control test (P < 0.0001 and F = 31.79, one-way ANOVA). Perfusion with vehicle (DMSO 10 μl/dl) for 15 min did not significantly alter coronary flow (5.39 ± 0.62 ml/min compared to basal value 5.40 ± 0.43 ml/min); in the presence of vehicle, 5-HT increased coronary flow to 9.84 ± 2.11 ml/min at 0.3 μM and 10.09 ± 1.05 ml/min at 1 μM (both P < 0.005 compared to the vehicle treatment). 5-HT could still elicit coronary flow increases after treatment of penitrem A 1 μM (P < 0.0001 and F = 13.48, one-way ANOVA). The basal coronary flow after 20 min re-equilibration was 5.36 ± 0.72 ml/min, and perfusion with penitrem A 1 μM for 15 min did not significantly alter coronary flow (6.16 ± 1.26 ml/min). In the presence of penitrem A, 5-HT at concentrations of 0.3 and 1 μM increased coronary flow to 10.25 ± 2.70 and 10.49 ± 2.35 ml/min, respectively (both P < 0.01 compared to penitrem A treatment). Since coronary flow responses to 5-HT were not significantly diminished by penitrem A, we did not perform histamine test at the end of experiment.

5-HT-induced coronary flow responses in L-NAME treated hearts did not significantly alter in the presence of paxilline 2 μM (Fig. 5b). In the control test, 5-HT induced significant coronary flow increases in the presence of vehicle (n = 5; P < 0.0001 and F = 45.88, one-way ANOVA). Treatment of vehicle (DMSO 10 μl/dl) did not significant alter coronary flow (6.79 ± 0.82 ml/min compared to the basal value 6.84 ± 1.14 ml/min); coronary flow increased to 15.29 ± 1.06 and 13.39 ± 2.32 ml/min after treatments of 5-HT 0.3 μM and 1 μM, respectively (both P < 0.001 compared to the vehicle treatment). Treatment of paxilline 2 μM slightly increased coronary flow to 8.45 ± 1.75 ml/min from the basal value 7.13 ± 1.03 ml/min; in the presence of paxilline 2 μM, 5-HT induced coronary flow increases to 14.94 ± 3.16 ml/min at 0.3 μM and 13.60 ± 2.79 ml/min at 1 μM (P < 0.01 and P < 0.05 compared to treatment of paxilline 2 μM, respectively).

As shown in Fig. 5c, treatment of TEA 300 μM significantly suppressed 5-HT-induced coronary flow increases (n = 5; P = 0.0014 and F = 22.67, 2-ways ANOVA). In the control test, 5-HT induced significant coronary flow increases (P < 0.0001 and F = 24.79, one-way ANOVA); the basal value of coronary flow was 5.03 ± 0.48 ml/min, and coronary flow after 15 min of prolonged perfusion was 4.78 ± 0.51 ml/min. 5-HT induced coronary flow increases to 9.32 ± 1.59 ml/min at 0.3 μM and 9.01 ± 1.37 ml/min at 1 μM (both P < 0.001 compared to the vehicle treatment point). 5-HT-induced coronary flow increase responses at 0.3 and 1 μM were suppressed in the presence of TEA 300 μM (P = 0.0632 and F = 2.970, one-way ANOVA). The basal coronary flow after re-equilibration was 4.28 ± 0.46 ml/min. After perfusion with TEA 300 μM for 15 min, coronary flow was 4.29 ± 0.68 ml/min. In the presence of TEA, 5-HT failed to cause significant coronary flow increases at 0.3 (5.24 ± 0.83 ml/min) and 1 μM (5.20 ± 0.78 ml/min). Histamine increased coronary flow to 11.76 ± 3.01 ml/min in the histamine test at the end of the experiment.

## Discussion

The vasorelaxation effect of 5-HT on human coronary arteries has been identified for more than 20 years [1, 2], but this property of 5-HT has not been addressed as much as the vasoconstrictive effect. 5-HT-induced coronary artery dilation has been reported to be NO-dependent in many species [13, 35, 36]; however, the 5-HT-induced coronary flow increases are, at least partially, resistant to L-NAME in rats [9]. In the present study, we investigated the role of 5-HT_7_ receptor in 5-HT-induced coronary flow increases in isolated rat hearts in the absence of L-NAME. As shown in Fig. 2b and c, both 5-HT and the selective 5-HT_7_ receptor agonist 5-CT [24] induced coronary flow increases in the absence of L-NAME, and both of these effects were blocked by 0.3 μM SB269970, which is a potent and selective 5-HT_7_ receptor antagonist (Ki value 1.26 nM [21]); furthermore, 5-HT turned to decrease coronary flow in the presence of SB269970, and this effect was blocked by the selective 5-HT_2A_ receptor antagonist R96544 0.3 μM (Fig. 2b). It is noteworthy that SB269970 blocked 5-CT-induced coronary flow increases, but 5-CT, which has very low affinity to 5-HT_2A_ receptor (Ki value 633–2700 nM) [24], did not cause coronary flow decrease in the presence of SB269970 (Fig. 2c). These results indicate that the coronary flow-decreasing component of 5-HT-induced coronary flow responses is mediated by 5-HT_2A_ receptor, which is in consensus with previous reports [37, 38], and both NO-dependent [11, 12, 38] and L-NAME-resistant [9] components of coronary flow increases induced by 5-HT at doses of 0.3 and 1 μM are mediated by the activation of 5-HT_7_ receptor.

Endothelium is an important apparatus in vascular tissues, and damage or malfunction of endothelium causes poor regulation of vascular responses. Endothelium helps regulate vessel tone by releasing vasoconstrictive agents [39] and vasodilating factors. NO [13], PGI_2_ [26], and EDHF [17] are the main dilating factors released from endothelium. NO induces an increase of cGMP in smooth muscle cells and then causes smooth muscle relaxation [40, 41]. PGI_2_ is a metabolite of COX [42]; it activates PGI_2_ receptors on smooth muscle cells, and then causes vasodilation by increasing intracellular cAMP [43]. PGI_2_ has been reported to mediate 5-HT-induced coronary vascular resistance reduce in rat hearts [14], but in the present study we did not find role of COX metabolites in 5-HT-induced coronary flow increases in L-NAME-treated hearts (Fig. 3b).

EDHF elicits vasodilation by inducing hyperpolarization of membrane potentials, which consequently prevents the opening of Ca^2+^ channels and hence reduces Ca^2+^ influx [17]. So far, there is no consensus on what the entity of EDHF is, and the exact mechanism(s) of how EDHF induces vasodilation remains controversial [18]; but the involvements of K^+^ ion and Ca^2+^-activated K^+^ channels in EDHF-induced vasorelaxation are commonly recognized [16]. Several models describing how Ca^2+^-activated K^+^ channels and K^+^ ion induce smooth muscle relaxation have been proposed [16–18]: One model suggests that the activation of Ca^2+^-activated K^+^ channel(s) on endothelium leads to K^+^ ion efflux, and the efflux of K^+^ ion causes membrane potential hyperpolarization of the endothelial cells, which then results in the membrane potential hyperpolarization of smooth muscle cells via gap junctions connecting the endothelium cells and the smooth muscle cells [44, 45]. In evidences for a second model, the released K^+^ ion from endothelium cells via Ca^2+^-activated K^+^ channel(s) increases local concentration of K^+^ ion in the intercellular space between the endothelium and the smooth muscle [46]; the elevated concentration of extracellular K^+^ in the loci causes the activation of inward rectified K^+^ channels or/and Na^+^/K^+^ pump on the smooth muscle cells [17] and hence induces the hyperpolarization of membrane potentials, which consequently prevents the vessels from constriction.

All 3 types of Ca^2+^-activated K^+^ channels have been reported involved in the EDHF-mediated vasorelaxation. For example, H_2_O_2_, one of the supposed EDHFs [47], is released from the endothelium in response to shear force and then causes dilation of human coronary arterioles by activating large-conductance Ca^2+^-activated K^+^ channel on the smooth muscle cells [48]. Small- and intermediate-conductance Ca^2+^-activated K^+^ channels, both of which can be activated by the arachidonic acid metabolites synthesized by cytochrome P450s and 15-LOX [18], have been reported mediating acetylcholine-induced vasodilation on coronary arterioles derived from normal rats [49], while large-conductance Ca^2+^-activated K^+^ channel plays no role in this response; this finding also implies that all these 3 types of Ca^2+^-activated K^+^ channels are not necessarily involved in an EDHF-mediated vasorelaxation at the same time.

Arachidonic acid metabolites-induced activation of Ca^2+^-activated K^+^ channels has been reported involved in EDHF-mediated vasorelaxation [15, 25]. In the present study, we investigated the role of EDHF in 5-HT-induced coronary flow increases by using pharmacological tools that interfere with the PLA_2_ - cytochrome P450 - Ca^2+^-activated K^+^ channel axis [18]. As shown in Fig. 3a-c, the inhibitions of PLA_2_ by quinacrine [15, 25] and cytochrome P450s by SKF525A [25] significantly suppressed 5-HT-induced coronary flow increases while the inhibition of COX by indomethacin [26] failed to influence the 5-HT-induced responses; these results indicate the involvement of the cytochrome P450s-synthesized arachidonic acid metabolites rather than PGI_2_ [14] in 5-HT-induced coronary flow increases in L-NAME-treated hearts. And as shown in Fig. 4a and b, blocking intermediate- and small-conductance Ca^2+^-activated K^+^ channels with TRAM-34 [30] and UCL1684 [31] significantly inhibited 5-HT-induced coronary flow increases in L-NAME-treated hearts. These results are in consensus with previous reports that epoxyeicosatrienoic acid isomers activate intermediate- and small-conductance Ca^2+^-activated K^+^ channels in EDHF-induced relaxation of the vascular tissues [18, 50, 51].

Activation of large-conductance Ca^2+^-activated K^+^ channel mediates vasorelaxation in various vascular tissues. For example, applications of BMS191011 and NS1619, both of which are openers of large-conductance Ca^2+^-activated K^+^ channel [52, 53], elicit vasodilation of isolated perfused sheep coronary arteries with pD2 (the negative logarithm to base 10 of the EC_50_) of 5.76 ± 0.4 and 5.86 ± 0.5, respectively [54]; in mice, large-conductance Ca^2+^-activated K^+^ channel is involved in acetylcholine-elicited NO-independent vasorelaxation on isolated skeletal muscle arterioles from diet-induced obese mice, although large-conductance Ca^2+^-activation K^+^ channel blocker does not affect the acetylcholine-induced response on the skeletal muscle arterioles from normal mice [49].

In the present study, the results are diverse in the experiments using the blockers to verify the role of large-conductance Ca^2+^-activated K^+^ channel in 5-HT-induced coronary flow increases in L-NAME-treated hearts (Fig. 5). Paxilline [33] and penitrem A [32] are both selective and potent large-conductance Ca^2+^-activated K^+^ channel blockers; TEA, the other blocker of large-conductance Ca^2+^-activated K^+^ channel we used, is generally used as non-selective K^+^ channels blocker [55], but at doses lower than 1 mM it is selective to large-conductance Ca^2+^-activated K^+^ channel [55]. As shown in Fig. 5a-c, paxilline 2 μM and penitrem A 1 μM failed to inhibit 5-HT-induced coronary flow increases in L-NAME treated hearts; however, 5-HT-induced coronary flow increases were blocked by TEA 300 μM. It is an interesting finding because in previous reports paxilline 0.2 μM is as potent as TEA 1 mM in blocking iberiotoxin-sensitive K^+^ current in isolated rat aorta smooth muscle cells [56], and in the smooth muscle cells isolated from rat vas deferens, paxilline 1 μM completely blocks large-conductance Ca^2+^-activated K^+^ channel current, which effect is similar to TEA 0.3 mM [57].

Penitrem A is a potent and selective large-conductance Ca^2+^-activated K^+^ channel blocker with IC_50_ of 6.40 nM [32], and it has been used to investigate the role of large-conductance Ca^2+^-activated K^+^ channel in vasorelaxation in several studies [32, 58, 59]. In porcine coronary endothelial cells, penitrem A 10 nM completely blocks the hyperpolarization elicited by 10 μM 1-EB10, a selective opener of large-conductance Ca^2+^-activated K^+^ channel [60], and partially inhibits the membrane potential hyperpolarization elicited by the adenosine receptor agonist 5′-ethylcarboxamidoadenosine 10 μM [61]; in portal vein smooth muscle cells from rats, treatment of penitrem 100 nM completely blocks the hyperpolarization elicited by NS1619 33 μM [53]. Paxilline is a potent and selective blocker of large-conductance Ca^2+^-activated K^+^ channel with Ki value 1.9 nM [33]. In rat isolated aortic smooth muscle cells, paxilline 1 μM almost completely blocks iberiotoxin-sensitive current with IC_50_ of 97 nM, while TEA blocks the iberiotoxin-sensitive current with IC_50_ of 273 μM [56]; in rat mesenteric arterial cells, paxilline completely blocks iberiotoxin-sensitive current at 300 nM [62]; in the isolated human coronary arterioles, paxilline 0.1 μM significantly inhibits H_2_O_2_-elicited vasodilation [48]. Judging from these studies, the failures of paxilline and penitrem A in inhibiting 5-HT-induced coronary flow increases in L-NAME treated hearts do not come from that the concentrations we used are not high enough since the doses we used in the present study (paxilline 2 μM and penitrem A 1 μM) are much higher than those in the aforementioned studies. Besides, according to our unpublished observation, the opener of large-conductance Ca^2+^-activated K^+^ channel BMS191610 does not elicit coronary flow increase at 3 and 10 μM in L-NAME-treated hearts (data not shown), both of which doses are higher than the EC_50_ of BMS191610 in inducing vasorelaxation of isolated normal fetal sheep coronary arteries in previous study [54].

Although evidences from paxilline and penitrem A do not support the involvement of large-conductance Ca^2+^-activated K^+^ channel in 5-HT-induced coronary flow increases in L-NAME treated hearts, coronary flow-increasing effect of 5-HT was suppressed by TEA (Fig. 5). As mentioned above, TEA is a widely used non-selective antagonist of various K^+^ channels, including 3 types of Ca^2+^-activated K^+^ channels, voltage-dependent K^+^ channel, ATP-sensitive K^+^ channel, and inward rectified K^+^ channels [55]. The suppressing effect of TEA on 5-HT-induced coronary flow increases in L-NAME-treated hearts did not likely come from blocking small- and/or intermediate-conductance Ca^2+^-activated K^+^ channels, because at doses lower than 1 mM TEA is selective to large-conductance Ca^2+^-activated K^+^ channel [63], and in our preliminary tests TEA 0.3 mM does not have significant effect on the coronary flow increases induced by SKA-31, a selective opener of small- and intermediate-conductance Ca^2+^-activated K^+^ channels [30], in L-NAME treated hearts (data not shown).

The evidences we mentioned above suggest that the difference between effects of TEA and the other two blockers on 5-HT-induced coronary flow increases does not likely come from failures of paxilline and peintrem A in blocking large-conductance Ca^2+^-activated K^+^ channel, but unfortunately we do not know what is the origin of the difference so far. The role of large-conductance Ca^2+^-activated K^+^ channel and/or the TEA-sensitive component in 5-HT-induced coronary flow increases in L-NAME treated hearts need further investigation.

## Conclusion

In conclusion, the activation of 5-HT_7_ receptor mediates 5-HT-induced coronary flow increases in isolated hearts in the absence of L-NAME as in L-NAME-treated hearts as previously reported [9]. The coronary flow-increasing effect of 5-HT in L-NAME-treated hearts resemble the characters of EDHF-induced vasorelaxation: the inhibitors of PLA_2_ and cytochrome P450s, but not the inhibitor of COX, block 5-HT-induced coronary flow increases in L-NAME treated hearts, and small- and intermediate-conductance Ca^2+^-activated K^+^ channels are also involved in the 5-HT-induced coronary flow responses in L-NAME treated hearts. However, the role of large-conductance Ca^2+^-activated K^+^ channel in 5-HT-induced coronary flow increases needs further investigation.

To the best of our knowledge, the present study is the first to investigate the role of EDHF in 5-HT-induced vasorelaxation on the coronary arteries.

## Abbreviations

5-CT: 5-carboxamidotryptamine
5-HT: 5-hydroxytryptamine
COX: Cyclooxygenase
DMSO: Dimethyl sulfoxide
EC50: Half maximal effective concentration
EDHF: Endothelium derived hyperpolarizing factor
IC50: Half maximal inhibitory concentration
L-NAME: Nω-Nitro-L-arginine methyl ester hydrochloride
LOX: Lipoxygenase
NO: Nitric oxide
NOS: Nitric oxide synthase
pD_2_: Negative logarithm to base 10 of the EC_50_
